# Twice-a-day exercise increases acute *MCT1* gene expression in skeletal muscle but does not change the lactate curve after 3 weeks of training in adult men

**DOI:** 10.1590/1414-431X2025e14500

**Published:** 2025-10-17

**Authors:** A.P. Arcoverde-Mello, V.A. Andrade-Souza, S.K. Learsi, F. Tomazini, T. Ataide-Silva, J. Kuang, R. Bertuzzi, C.G. Leandro, D.J. Bishop, A.E. Lima-Silva, T. Ghiarone, K.A.S. Silva

**Affiliations:** 1 Grupo de Pesquisa em Ciências do Esporte, Universidade Federal de Pernambuco, Recife, PE, Brasil; 2 Programa de Pós-Graduação em Nutrição, Universidade Federal de Pernambuco, Recife, PE, Brasil; 3 Grupo de Pesquisa em Desempenho Humano, Universidade Tecnológica Federal do Paraná, Curitiba, PR, Brasil; 4 Grupo de Pesquisa em Ciências Aplicadas ao Esporte, Instituto de Ciências Biológicas e da Saúde, Universidade Federal de Alagoas, Maceió, AL, Brasil; 5 Programa de Pós-Graduação em Nutrição, Universidade Federal de Alagoas, Maceió, AL, Brasil; 6 Institute for Health and Sport, Victoria University, Melbourne, Victoria, Australia; 7 Grupo de Estudo em Desempenho Aeróbio, Escola de Educação Física e Esporte, Universidade de São Paulo, São Paulo, SP, Brasil; 8 Department of Biomedical Sciences, Cooper Medical School of Rowan University, Camden, NJ, USA; 9 Grupo de Estudos e Pesquisas em Fisiologia Humana e do Exercício, Instituto Federal de Alagoas, Maragogi, AL, Brasil

**Keywords:** Monocarboxylate transporters, Lactate slope index, Exercise training, Low carbohydrate training

## Abstract

We have previously demonstrated that different modalities of endurance exercise combined with lower muscle glycogen content elicit several physiological and molecular benefits in men. In this study, we hypothesized that these exercise strategies modulate monocarboxylate transporters (MCTs) and plasma lactate. We investigated *MCT1* and *MCT4* gene expression after two forms of exercise (i.e., once daily and twice-a-day) under low carbohydrate (CHO) availability (Acute - Study 1) and whether three weeks under once daily or twice-a-day training differentially affected plasma lactate during exercise (Chronic - Study 2). In Study 1, five participants performed a high-intensity interval exercise (HIIE) 2 h (twice-a-day) or 15 h (once daily) after exercise and diet manipulations to reduce endogenous CHO stores or without previous CHO manipulation (Control). Muscle biopsies were collected before, right after, and 3 h after HIIE. In Study 2, plasma lactate was measured during a graded exercise test before and after three weeks of once-daily (n=7) or twice-a-day training (n=7). *MCT1* gene expression increased from before to after and 3-h post-HIIE only in the twice-a-day exercise (P<0.05). *MCT4* gene expression was unaltered in all conditions (P>0.05). The plasma lactate curve shifted to the right in both training approaches, without differences in lactate slope reduction between once-daily (-0.49±0.58 mmol·L^-1^·min^-1^) and twice-a-day (-0.46±0.73 mmol·L^-1^·min^-1^) exercise. In conclusion, twice-a-day training increased acute *MCT1* gene expression but did not result in chronic changes in plasma lactate response during exercise.

## Introduction

Lactate produced in the skeletal muscle is transported through membranes via monocarboxylate-transporting proteins (MCTs). Two main MCT isoforms are present in skeletal muscle: MCT1 and MCT4 (1-4). The MCT1 protein is more responsive to endurance training than MCT4 (5,6). Accordingly, MCT1 protein content might contribute to the well-documented reduction in plasma lactate concentration during exercise after endurance training (7). An increase in MCT protein content after endurance training might result from an accumulative increase in *MCT* gene expression that occurs after every single endurance exercise session (8). The *MCT1* gene expression (also known as SLC16A1) is also greater than that of *MCT4* (also known as SLC16A3) after a single endurance exercise session (9). Thus, evaluating new training strategies to optimize *MCT* gene expression might contribute to a better understanding of the potential link between *MCT* gene expression and training-induced changes in plasma lactate.

One strategy to manipulate exercise-induced gene expression is to combine endurance exercise with low carbohydrate (CHO) availability, the so-called “train-low” strategy (10). However, whether the “train-low” strategy regulates *MCT1* and *MCT4* gene expression is unclear. Although there are different “train-low” approaches (11), the two most common are: 1) “twice-a-day” training, in which the first exercise session reduces muscle glycogen stores and a second exercise session is performed 1 to 3 h later, with reduced initial muscle glycogen stores; and 2) “sleep-low” or “once-daily” training, in which the first exercise session to reduce muscle glycogen content is performed in the evening, followed by fasting or CHO intake restriction until a second training session the next day (∼11 to 14 h interval between the two training sessions). In both training modalities, the second exercise session will start with reduced muscle glycogen stores (11). While these two strategies seem to activate several genes associated with mitochondrial biogenesis, a study demonstrated that, in comparison with the acute once-daily training, acute twice-a-day training potentiated the transcription of peroxisome proliferator-activated receptor-γ coactivator 1-alpha (*PGC-1α*), peroxisome proliferator-activated receptor-alpha (*PPARα*), and peroxisome proliferator-activated receptor beta/delta (*PPARβ/δ*) genes (12). Thus, twice-a-day training might be a promising exercise strategy for optimizing the activation of genes associated with mitochondrial biogenesis, particularly *PGC-1α* (considered a key regulator of mitochondrial biogenesis (13).

Interestingly, a positive association has been reported between PGC-1α and MCT1 protein content in rat hind limb muscles (14). In addition, lactate acts as a signaling molecule in L6 cells, increasing both *MCT1* and *PGC1α* gene expression (15). Although these studies do not provide evidence that *MCT1* is directly regulated by *PGC-1α*, it could be suggested that the lactate signaling cascade might converge on transcription factors affecting *MCT1* and *PGC1α* gene expression. As *PGC1α* gene expression is higher in the twice-a-day than once-daily training session, exercise-induced *MCT1* gene expression might also be higher after a single session of twice-a-day than a once-daily approach. To date, however, no study has tested this hypothesis, which could provide important insights regarding molecular signaling induced by twice-a-day and once-daily training approaches.

Another assumption is that, as training-induced adaptations have been proposed to be associated with cumulative, transient increases in mRNA expression (16), a potential rise in *MCT1* gene expression with successive twice-a-day exercise bouts might influence the exercise-induced increase in plasma lactate after a training period. While the link between changes in MCT protein content and lactate transport capacity in response to training is still controversial (for review, see reference 5), it would be interesting to investigate whether two types of low-train strategies that are expected to result in different acute *MCT1* gene expression also result in a different exercise-induced increase in plasma lactate after a training period. However, whether twice-a-day and once-daily training differentially affects the training-induced changes in the plasma lactate response to exercise is currently unknown. As training-induced changes in the plasma lactate response to exercise have been classically investigated during a graded exercise test (17,18), it is of interest to understand whether the plasma lactate curve during a graded exercise test is affected differently by twice-a-day and once-daily training.

Therefore, the first aim of the present study was to quantify the acute gene expression of *MCT1* and *MCT4* after a single session of the twice-a-day or once-daily approach (Study 1). We hypothesized that the twice-a-day approach would result in a greater increase in the muscle expression of *MCT1* and *MCT4* compared to the once-daily approach. The second aim was to investigate whether three weeks of twice-a-day or once-daily training differentially affects the plasma lactate curve during a graded exercise test (Study 2). We hypothesized that three weeks of once-daily and twice-a-day training would shift the plasma lactate curve differently during a graded exercise test.

## Material and Methods

### Participants

This study is part of an umbrella project in which other aspects of the project were published elsewhere (12,19). Samples from 5 random participants were extracted from the first study to analyze the gene content of *MCT1* and *MCT4*. In the second study, 15 healthy and physically active men were recruited and randomized into the twice-a-day and once-daily groups, but one participant was excluded from the analyses because he was considered a true outlier in relation to lactate parameters (Grubbs' test, also called the extreme studentized deviate ESD method, with prism outlier calculator, alpha=0.05) in the second study. As the data reported here were *a posteriori* data, sample size calculation was done based on the primary outcomes detailed and documented in previous publications by our group (12,19). The main morphological and physiological characteristics of the participants are described in Tables 1 and 2. The participants were recruited from March 15, 2015 to October 31, 2016 through online advertisements and folders on the university campus (Federal University of Pernambuco). The following inclusion criteria were used: 1) physically active (exercising more than 3 times per week) and 2) habituated to cycling. The following exclusion criteria were adopted: 1) history or signs of cardiac, metabolic, or respiratory diseases and/or 2) use of any prescription medications. Participants were informed about the risks and benefits inherent to the experiments. All participants signed a written consent before enrolling in the study, which was conducted following the principles of the Declaration of Helsinki and approved by the Research Ethics Committee of the Federal University of Pernambuco (CAAE: 30378414.8.0000.5208).

**Table 1:** Main characteristics of the participants in Study 1 (n=5).

Variables	Mean±SD
Age	33.2±1.9
Weight (kg)	78.6±9.9
Height (m)	1.76±0.07
Body fat (%)	13.6±5.1
Exercise duration (min)	34.6±3.1
PPO (W)	229.6±38.2
V̇O_2peak_ (mL·kg^-1^·min^-1^)	37.1±6.4
[La]_peak_ (mmol/L)	9.7±1.8
HR_peak_	177±15

Data are reported as means±SD. PPO: peak power output; V̇O_2peak_: peak oxygen uptake; [La]_peak_: peak plasma lactate concentration; HR_peak_: peak heart rate.

**Table 2:** Main characteristics of the participants Study 2 (n=14).

Variables	Groups		
	Once-daily (n=7)	Twice-a-day (n=7)	P value
Age	29.1±5.1	24.4±9.3	0.071
Weight (kg)	72.5±7.0	81.1±12.8	0.126
Height (m)	1.80±0.01	1.79±0.08	0.930
Body fat (%)	13.3±4.8	13.6±4.4	0.788
Exercise duration (min)	41.6±4.4	42.4±7.6	0.822
PPO (W)	235.1±21.8	237.0±36.1	0.559
V̇O_2peak_ (mL·kg^-1^·min^-1^)	38.1±6.6	35.6±3.9	0.216
[La]_peak_ (mmol/L)	10.4±1.3	9.3±2.6	0.350
HR_peak_	182±9	181±9	0.832
Parameter “a” (mmol/L)	1.28±0.63	1.31±1.05	0.957
Parameter “b” (mmol·L^-1^·min^-1^)	0.60±0.59	0.56±0.75	0.920
Parameter “c” (units)	0.07±0.01	0.10±0.08	0.357

Data are reported as means±SD. Parameters “a”, “b”, and “c” were determined using the least squares method for an exponential equation, where “a” is the intercept, “b” is the scaling coefficient (i.e., lactate slope index), and “c” is the rate constant. PPO: peak power output; V̇O_2peak_: peak oxygen uptake; [La]_peak_: peak plasma lactate concentration; HR_peak_: peak heart rate.

### Experimental design

#### Study 1

**Figure 1:**
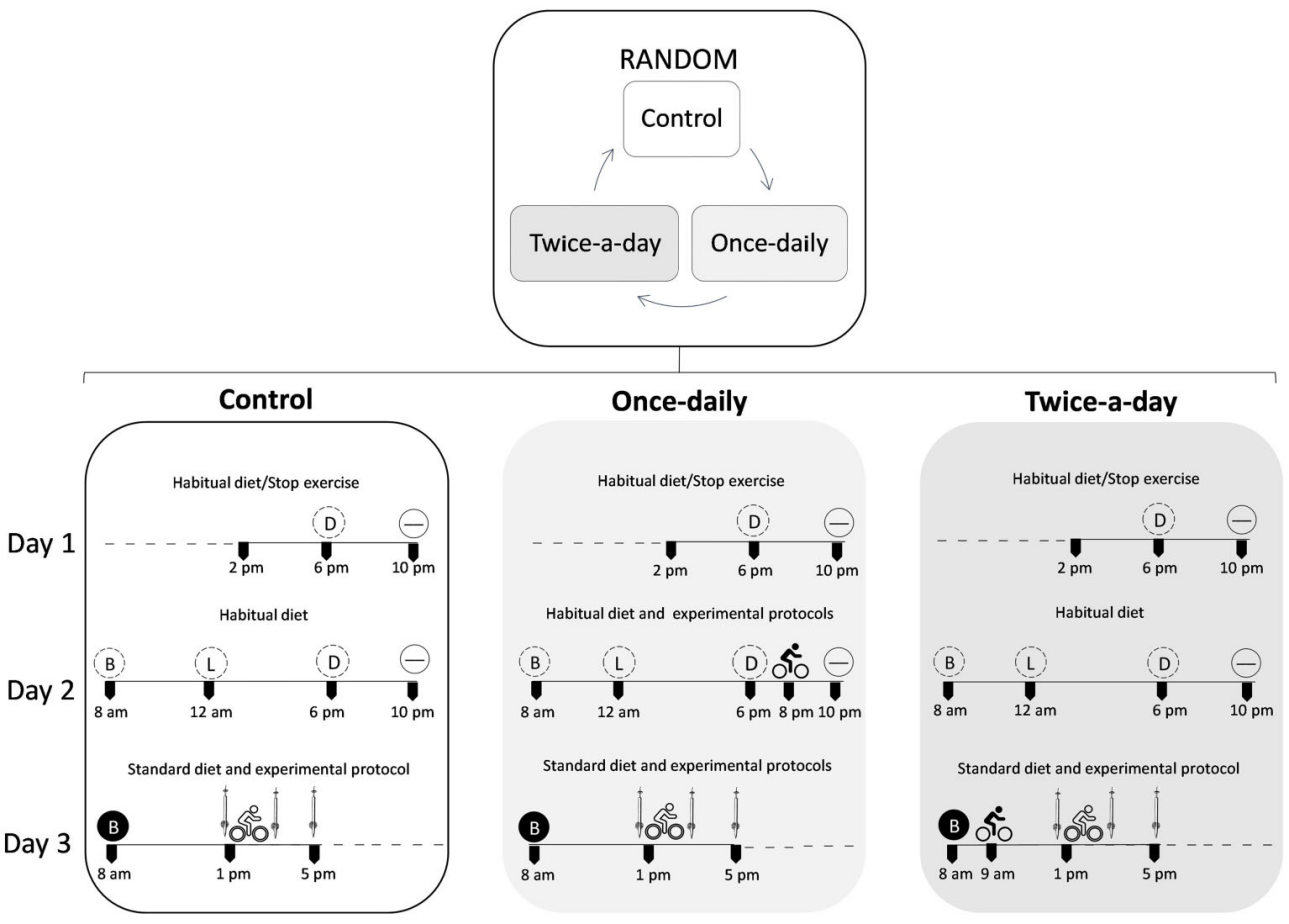
Design overview of Study 1. Habitual diet: 65% carbohydrate, 10-35% protein, and 20-35% lipids. Low-carbohydrate diet: ∼7% carbohydrate; ∼33% protein; ∼60% lipids. B: breakfast; L: lunch; D: dinner.

The participants were first subjected to anthropometric measurements (height, weight, and body fat percentage) and completed a graded exercise test to determine their peak oxygen consumption (V̇O_2peak_), peak power (PPO), and the first and second lactate thresholds, which were further used to determine the exercise workload of the experimental trials. Participants completed three experimental trials in a randomized, crossover design. An overview of the experimental design is shown in Figure 1. On day 1 at 8:00 PM (the day before the experimental trial), participants in the once-daily group performed a 100-min endurance exercise + six supramaximal sprints to deplete muscle glycogen stores. After overnight fasting, participants ate a low-CHO breakfast (∼7% CHO) at 8:00 AM and performed a HIIE at 1:00 PM on the trial day. In the twice-a-day group, on the trial day, participants ate a low-CHO breakfast (∼7% CHO) at 8:00 AM and performed the endurance exercise + supramaximal sprints and the HIIE at 9:00 AM and 1:00 PM, respectively. In the control group, participants ate a low-CHO breakfast (∼7% CHO) at 8:00 AM and performed the HIIE at 1:00 PM on the trial day without performing the muscle glycogen depletion protocol. Skeletal muscle biopsies from the vastus lateralis were taken before, immediately after, and 3 h after completion of the HIIE. In all experimental trials, participants were advised to refrain from food between breakfast and the last muscle biopsy.

#### Study 2

**Figure 2:**
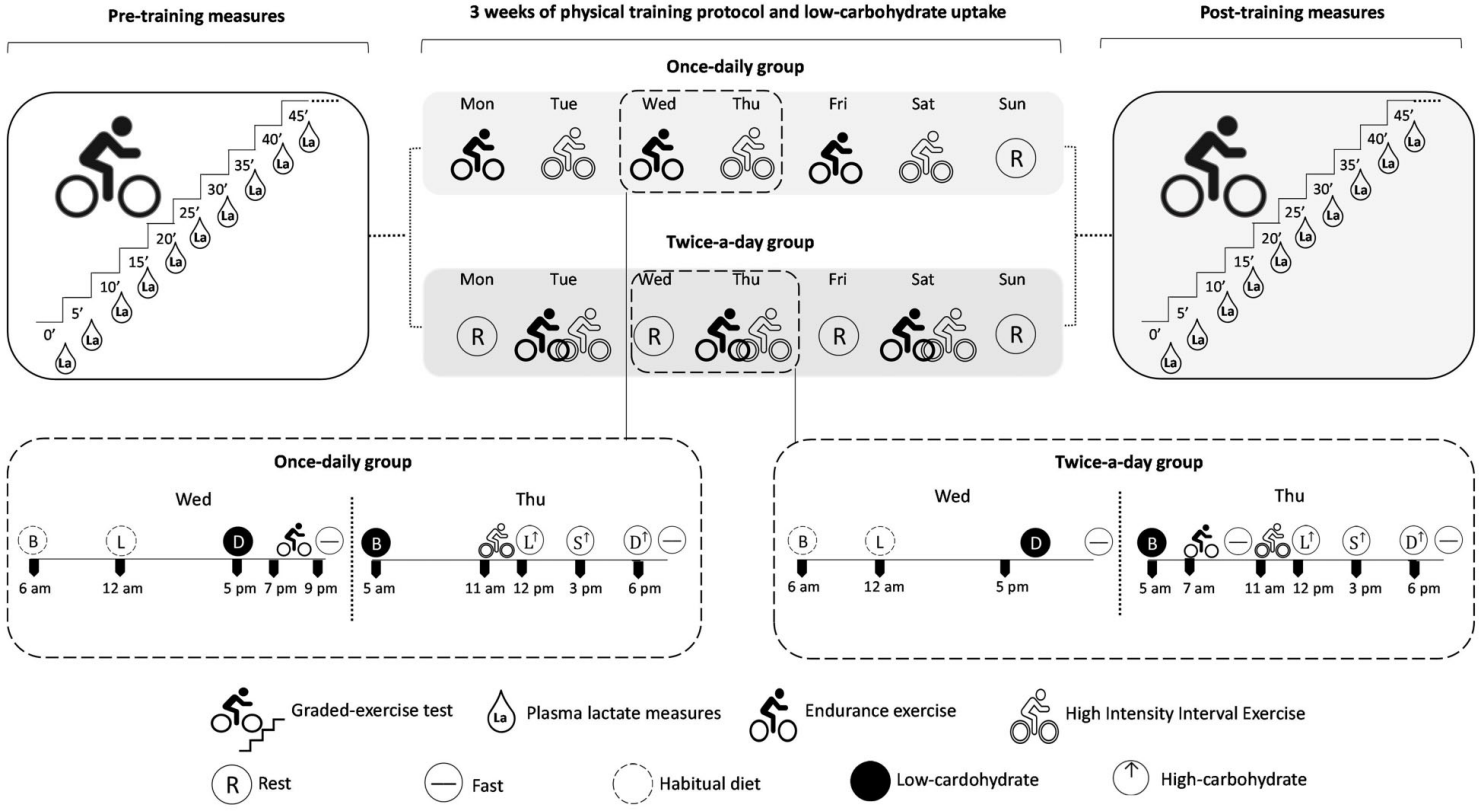
Design overview of Study 2. R: rest; B: breakfast; L: lunch; D: dinner; S: snack. Habitual diet: 65% carbohydrate, 10-35% protein, and 20-35% lipids. Low-carbohydrate diet: ∼7% carbohydrate, ∼33% protein, and ∼60% lipids; High-carbohydrate diet: 75% carbohydrate, 10% lipids, and 15% proteins.

Fourteen volunteers were randomly allocated into one of two groups: once-daily (n=7) or twice-a-day training (n=7). Figure 2 shows an overview of the experimental design. Briefly, in the first visit, participants performed a graded exercise test to determine their pre-training plasma lactate curve profile. Forty-eight hours later, participants returned to the laboratory to familiarize themselves with the training protocol. Participants then completed a 3-week training program. The training protocol consisted of an initial training session consisting of an endurance exercise and a second training session consisting of an HIIE. Participants in the once-daily group trained six days a week, with the first training session (endurance exercise) performed in the evening (8:00 PM) and the second training session (HIIE) in the morning (11:00 AM) of the next day (15 h between training sessions). Participants in the twice-a-day group trained three times a week and performed both training models in the same day, with endurance exercise starting at 7:00 AM and ending at 9:00 AM and HIIE at 11:00 AM (2 h between training sessions). Participants executed only the prescribed training and followed a strict dietary plan, as detailed elsewhere (19). Finally, participants performed a graded exercise test to determine their post-training plasma lactate curve profile 48 h after the last training session.

### Experimental procedures

#### Graded exercise test

The graded exercise test was performed on an electromagnetically-braked cycle ergometer (Ergo-Fit 167, Germany). The test started at 50 W and then increased the work rate by 25 W every 4 min, with a 1-min break between stages. The participants maintained the pedal cadence at ∼70 rpm throughout the test. The test was interrupted when the participant could no longer hold the required pedal cadence or voluntarily disengaged from exercise (20).

Participants used a facemask and O_2_ uptake (V̇O_2_), carbon dioxide production (V̇CO_2_), and pulmonary ventilation (V̇E) were recorded breath-by-breath using a metabolic cart (Cortex Metalyzer 3B, Cortex Biophysik, Germany). The O_2_ and CO_2_ concentrations were analyzed using electrochemical cell and infrared analyzers, respectively. The O_2_ and CO_2_ sensors were calibrated before each test using gases of known concentration (12% O_2_ and 5% CO_2_). The volume transducer was pre-calibrated using a 3-L syringe. The heart rate (HR) was recorded using an HR monitor (Polar T 31/34^®^, Finland). At the end of each 4-min bout, a capillary blood sample (40 μL) was also collected from the ear lobe for subsequent determination of plasma lactate concentration ([La]).

The V̇O_2peak_ was defined as the highest 30-s average V̇O_2_ during the test (21), while the PPO was defined as the highest workload reached during the test (22). If a participant did not complete the final stage (i.e., <4 min), PPO was determined using the fractional time in the last incomplete stage multiplied by the increment rate (22). To determine the workloads of both endurance and HIIE sessions, the first and second lactate thresholds were identified, as previously described (12). Briefly, the first lactate threshold was visually identified as the first increase in plasma lactate concentration above the resting level, and the second lactate threshold was identified by the modified Dmax method (23).

#### Endurance exercise

The endurance exercise consisted of 100 min of cycling at a work rate corresponding to 50% of the difference between the first and the second lactate threshold. After an 8-min rest, participants performed six 1-min bouts at 125% of PPO, with a 1-min rest between bouts. This exercise protocol reduces muscle glycogen stores by ∼45%, as previously reported (12).

#### High-intensity interval exercise

The HIIE consisted of a 5-min warm-up at 90% of the first lactate threshold, followed immediately by ten 2-min bouts at a work rate corresponding to 20% of the difference between the second lactate threshold and PPO, with a 1-min passive rest between bouts (20).

### Analysis

#### Muscle tissue samples and analysis (Study 1)

Muscle tissue was obtained via muscle biopsy, using a Bergström needle adapted for manual suction (24,25). Nine separate incisions (three per trial) were performed in the *vastus lateralis* under local anesthesia (2% xylestesin). Samples were taken approximately 1 cm apart from a previous biopsy site. Samples were immediately snap frozen in liquid nitrogen and then stored at -80°C until subsequent analyses of *MCT1* and *MCT4* gene expression.

#### Real-time quantitative PCR (Study 1)

The extraction and real-time analyses have previously been described in detail (12). Briefly, 10 to 15 mg of frozen muscle was homogenized in 800 µL of TRIzol reagent (Thermo Fisher Scientific, USA) using a TissueLyser II (Qiagen, Germany) to obtain the RNA (26). The concentration and purity of each sample were evaluated using a NanoDrop One/Onec (Thermo Fisher Scientific), and the RNA integrity of a subset of samples was measured using a Bio-Rad Experion microfluidic gel electrophoresis system with the Experion RNA StdSens analysis kit (Bio-Rad, 7 007 104, USA). Reverse transcription to cDNA was performed using a thermocycler (Bio-Rad) and iScriptRT Supermix (Bio-Rad, 170-8840) according to the manufacturer's instructions, in 1 µg of extracted RNA, in a total reaction volume of 20 µL. All samples were collected during the same run.

Relative mRNA expression was measured by qPCR (QuantStudio 7 Flex, Applied Biosystems, USA) using SsoAdvanced Universal SYBR Green Supermix (BioRad). Primers were designed using Primer-BLAST45 to include all splice variants and were purchased from Sigma-Aldrich, USA. All reactions were performed in duplicate on 384-well MicroAmp optical plates (Applied Biosystems) using an epMotion M5073 automated pipetting system (Eppendorf AG, Germany). The total reaction volume was 5 µL and contained 2 µL of diluted template cDNA, 2.5 µL of MasterMix, and 0.3 or 0.9 µM of primers. Gene stability was determined by BestKeeper46 NormFinder47 software, and the three most stably expressed genes were TATA box-binding protein (TBP). The expression of the target genes was calculated using the 2^-ΔΔCt^ method in relation to TBP expression. *MCT1* and *MCT4* primers used in this study were as follow: *MCT1* sense sequence 5′-TGACCATTGTGGAATGCTGT-3′ and antisense sequence 5′-GAGCCGACCTAAAAGTGGTG-3′; *MCT4* sense sequence 5′-GCATCTCCTACGGCATGGTG-3′ and antisense sequence 5′-CAGGAGTTTGCCTCCCGAA-3′; TBP sense sequence 5′-CAGTGACCCAGCAGCATCACT-3′ and antisense sequence 5′-AGGCCAAGCCCTGAGCGTAA-3′. Data of *MCT1* and *MCT4* are reported as fold changes from the Control group pre-HIIE.

#### Plasma lactate curve profile (Study 2)

Blood samples were transferred to microtubes containing 10 μL of EDTA and centrifuged at 1500 *g* for 10 min at 4°C (Hermle Labortechnik GmbH, Germany) for plasma separation from red and white cells. The plasma [La] was analyzed in duplicate in a spectrophotometer (Biospectro, SP184 22, Brazil) using a commercial kit (Labtest, Brazil). To determine the plasma lactate curve profile, the plasma [La] was plotted as a function of exercise time, and the curve was adjusted using an exponential function (Hughson et al. (27)): Equation 1: y = a + b exp (cx) + e, in which “y” is the plasma [La] in a given “x” time, “a”, “b”, and “c” are determined using the least squares method, “e” is the residual error, and “exp (cx)” is the maximized estimate of the correlation coefficient between x and y.

This continuous exponential model provides a good fit of the lactate-exercise time curve during a graded exercise test (27,28). In this model, a lactate slope index (parameter *b* from Equation 1) is used as a marker of aerobic fitness (28,29).

### Statistical analyses

Statistical analysis was performed using GraphPad Prism software version 8.02. Data were checked for normality using the Kolmogorov-Smirnov test, and outliers were detected with Grubbs' test. In Study 1, two-way repeated-measures ANOVA with condition (twice-a-day, once-daily, and control) and moment (pre-, post, and 3-h post-HIIE) as factors was used to compare *MCT1* and *MCT4* gene expression responses. In Study 2, pre-training differences between twice-a-day and once-daily groups were checked using an independent Student's *t*-test. A two-way mixed-model ANOVA was used to compare the parameters obtained from Equation 1, with group (twice-a-day and once-daily) and training (pre- and post-training) as factors. Partial eta squared (η_p_^2^) for main and interaction effects were calculated and interpreted as small (η_p_^2^<0.06), moderate (0.06≤η_p_^2^<0.15), or large (η_p_^2^≥0.15) (30). A Bonferroni *post hoc* test was used to locate differences when necessary. Data are reported as means±SD, and the significance level was set at P<0.05.

## Results

### Muscle *MCT* gene expression (Study 1)

**Figure 3:**
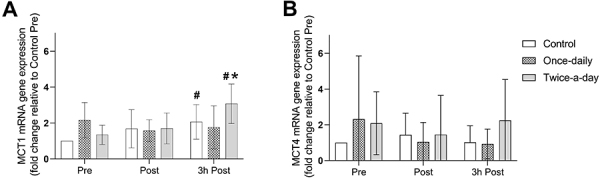
Monocarboxylates transport type 1 (*MCT1*) gene expression (**A**) and monocarboxylates transport type 4 (*MCT4*) gene expression (**B**) measured before (Pre), right after (Post), and 3 h after a high-intensity interval exercise. Data are reported as means±SD. ^#^P<0.05 compared to Pre and 3 h post only for the control and twice-a-day condition; *P<0.05 compared to the once-daily condition (two-way ANOVA).

There was a significant condition *vs* moment interaction for *MCT1* gene expression (F_(4,16)_=3.088, P=0.046, η_p_^2^=0.44, Figure 3A). The *MCT1* gene expression increased 3 h after the HIIE in the twice-a-day condition compared to pre- and post-HIIE time points (P=0.001 and 0.009). The *MCT1* gene expression 3 h post-HIIE was ∼80% higher in the twice-a-day than in the once-daily condition (P=0.038). In the once-daily condition, there were no changes in *MCT1* from pre- to post-HIIE or 3 h after the HIIE (P>0.05). In the control condition, the *MCT1* gene expression 3 h after the HIIE was higher than the corresponding pre-HIIE (P=0.046). There was no effect of condition (F_(2.8)_=0.437, P=0.660, η_p_^2^=0.10), moment (F_(2.8)_=0.526, P=0.610, η_p_^2^=0.12), or condition *vs* moment interaction (F_(4.16)_=1.491, P=0.252, η_p_^2^=0.27) for *MCT4* gene expression (Figure 3B).

### Lactate curve profile (Study 2)

There were no significant pre-training differences between the twice-a-day and once-daily groups for main participants' characteristics or for any parameter from Equation 1 (Table 2, P>0.05).

**Figure 4:**
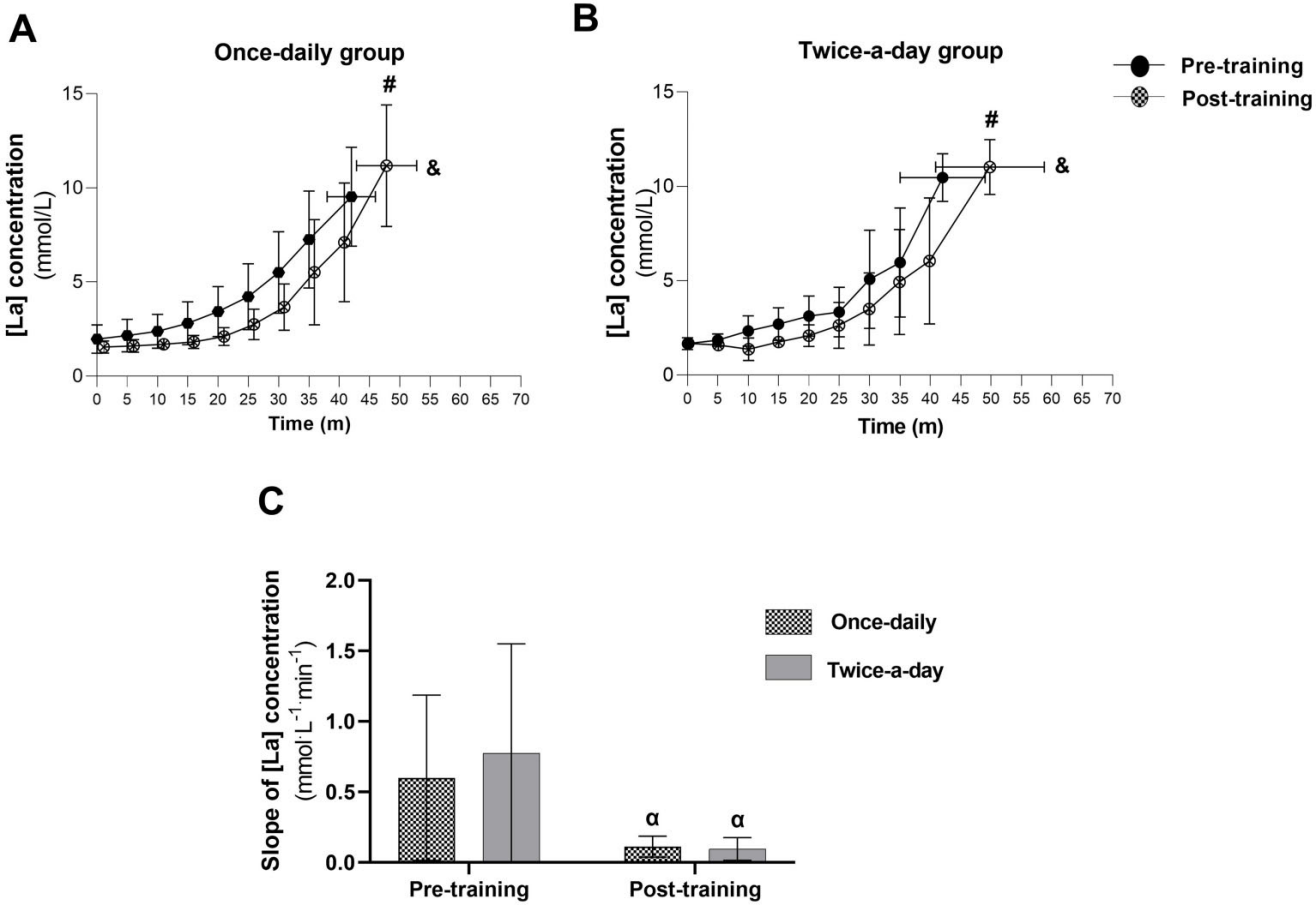
The effect of three weeks of once-daily (**A**) or twice-a-day training (**B**) on plasma lactate [La] concentration curve profile during a graded exercise test. The effect of the two training approaches on the lactate slope index is presented in **C**. Data are reported as means±SD. ^#^P<0.05, ^&^P<0.05, ^α^P<0.05 compared to pre-training in both groups (two-way ANOVA).

Figure 4A and B show the plasma lactate curve profile pre- and post-once-daily and twice-a-day training. Exercise duration and peak plasma [La] increased (main effect of time, F_(1,12)_=197.3 and 9.869, P<0.01, η_p_^2^=0.94 and η_p_^2^=0.45, Figure 4A and B) from pre- to post-training in both the once-daily (from 41.6±4.4 to 47.5±4.9 min and 10.4±1.3 to 11.0±1.5 mmol/L) and twice-a-day groups (from 42.4±7.6 to 49.5±8.7 min and 9.2±2.7 to 10.6±2.6 mmol/L). There was no main effect of group (F_(1,12)_=0.159 and 0.500, P=0.697 and 0.493, η_p_^2^=0.01 and η_p_^2^=0.04) or group *vs* time interaction (F_(1,12)_=1.941 and 1.300, P=0.189 and 0.276, η_p_^2^=0.14 and η_p_^2^=0.10).

The plasma lactate curve shifted to the right in both groups after training (Figure 4A and B). As a consequence, the lactate slope index (parameter *b* in Equation 1) was significantly reduced (main effect of time, F_(1,12)_=10.512, P=0.007, η_p_^2^=0.47) from pre- to post-training in both the once-daily (from 0.60±0.59 to 0.11±0.07 mmol·L^-1^·min^-1^) and twice-a-day groups (from 0.56±0.75 to 0.10±0.07 mmol·L^-1^·min^-1^, Figure 4C). There was no main effect of group (F_(1,12)_=0.171, P=0.687, η_p_^2^=0.01) or group *vs* time interaction (F_(1,12)_=0.275, P=0.609, η_p_^2^=0.02) for the lactate slope index. There was no main effect of group, time, or group *vs* time interaction for the other parameters of Equation 1 (P>0.05).

## Discussion

We assessed the gene expression of *MCT1* and *MCT4* after once-daily and twice-a-day exercises under low glycogen muscle content (Study 1) and the changes in the plasma lactate curve during a graded exercise test after three weeks of twice-a-day and once-daily training (Study 2). Findings of Study 1 showed that under low glycogen muscle content, twice-a-day, but not once-daily exercise, increased *MCT1* gene expression. However, despite the differences in exercise-induced *MCT1* gene expression, findings of Study 2 showed that both training strategies similarly shifted the plasma lactate curve to the right during a graded exercise test. Furthermore, based on their baseline V̇O_2peak_, participants were considered as having regular functional aerobic capacity (31), which is compatible with their classification as “physically active” but not athletes. Importantly, there was no significant difference in baseline V̇O_2peak_ between the two training groups, indicating that any difference between the groups for training responses was not caused by differences in aerobic fitness.

Partially corroborating our first hypothesis, the findings of Study 1 indicated that *MCT1* gene expression significantly increased with the control and twice-a-day approach but not with the once-daily approach. In contrast to *MCT1*, the expression of the *MCT4* did not significantly change under any condition. Although the reason for the reduced responsiveness of *MCT4* gene expression to acute exercise is unclear, this aligns with the conclusion of a recent review stating that the effects of acute exercise on *MCT1* and *MCT4* gene expression are less well-defined and that more research is needed, particularly in humans (32). Interestingly, gene expression of *MCT1* and *MCT4* did not increase immediately post-HIIE in any condition. Instead, gene expression of *MCT1* but not *MCT4* was increased at 3 h post-exercise in the twice-a-day and control, but not in the once-daily approach. These findings align with studies reporting that the detection of an increased mRNA content occurs approximately 3 h after an acute exercise (33). Furthermore, the fact that *MCT1* gene expression was increased in the control (∼106%) and twice-a-day (∼200%) conditions 3 h post-HIIE indicates that HIIE *per se* induces sufficient stimulus to transcribe *MCT1* mRNA. Although it was not significantly different from the control condition, the endurance exercise for muscle glycogen depletion in the twice-a-day condition notably exerted an additive effect on the *MCT1* gene expression (∼50% higher than control 3 h post-HIIE), likely due to the short interval of the combinatory stimulus of endurance and HIIE. Intriguingly, the once-daily condition did not modulate *MCT1* gene expression within the assessed time points, although it was ∼76% higher 3 h post-HIIE than pre-HIIE. Although we have not examined the level of *MCT1* gene expression shortly after the muscle glycogen depletion exercise (e.g., 3 h post-exercise), we speculate that, as endurance exercise in the once-daily condition was performed in the evening before, it is probable that the endurance exercise promoted an increase in *MCT1* expression overnight. In fact, it has been demonstrated that acute endurance exercise increases *MCT* gene expression (5). However, as ∼14 h separated endurance exercise and HIIE in the once-daily condition and *MCT1* protein expression is substantially suppressed 12 h after exercise (5), the HIIE stimulus might not have been sufficient to counteract the inhibited *MCT1* gene expression in the once-daily condition.

While no study has investigated the effect of the twice-a-day and once-daily approaches on *MCT1* and *MCT4* gene expression, previous research has identified some genes related to mitochondrial biogenesis in which the expression is potentiated by the twice-a-day approach, such as Peroxisome proliferator-activated receptor-alpha (*PPARA*) and *PGC1α* gene expression (12). In addition, it has been proposed that the lactate signaling cascade might converge on transcription factors affecting mitochondrial biogenesis (14,15). In this regard, lactate might act as a signaling molecule, which can regulate the expression of many genes (34,35), including *MCT1* (35,36). It is anticipated that both endurance exercise and HIIE performed in the present study increase plasma lactate to ∼4 mmol/L (19). Because the endurance exercise and the HIIE were performed only 2 h apart in the twice-a-day approach, this may have increased exposure to high plasma lactate levels. This exposure might have contributed to the high *MCT1* gene expression. In fact, *MCT1* gene expression was not altered in the once-daily approach, where endurance exercise and HIIE were performed many hours apart from each other. Thus, our findings suggest that the increased *MCT1* gene expression in the twice-a-day exercise may be linked with a prolonged exposure to elevated plasma lactate levels during this type of training, and that prolonged exposure to elevated plasma lactate may be necessary to promote increased *MCT1* gene expression. Together with previously reported findings (12), our results suggest that the twice-a-day approach might simultaneously provoke a greater increase in the expression of genes related to muscle mitochondrial biogenesis and lactate transport through sarcolemma than the once-daily approach. Although somewhat speculative, because we did not measure MCT1 protein content, it is plausible to hypothesize that as MCT1 facilitates lactate uptake and oxidation in cells with high mitochondrial densities (37), a long-term exposure to increased plasma lactate concentration might be a powerful stimulus to increase *MCT1* gene expression and MCT1 protein content.

The findings of Study 2 showed that the lactate slope index reduced similarly after three weeks of twice-a-day and once-daily training. It is well established that endurance training reduces the rate of plasma lactate accumulation during a graded exercise test, shifting the plasma lactate curve to the right (23,38). These changes in the plasma lactate curve are not greatly influenced by alterations in lactate production but are almost entirely attributed to the increased capacity to oxidize lactate, which is directly related to lactate removal from the muscle (36). In turn, increased MCT1 protein content is related to increased lactate removal (2). Thus, contrary to our second hypothesis, the increased *MCT1* gene expression found after a single session of twice-a-day training did not precede a greater attenuation of plasma lactate accumulation following three weeks of the twice-a-day training approach. Although a small increase in MCT1 protein content has been reported after a single session exercise (9), it is possible that three weeks of training may not have been sufficient to generate significant differences in MCT1 protein content between twice-a-day and once-daily approaches. Unfortunately, we were unable to measure pre- and post-training MCT1 protein content. Perhaps, a longer training period would increase the difference in the plasma lactate response between the two training approaches. In addition, there was considerable variability in the training-induced changes in plasma lactate response to exercise across individuals (data not shown), which may have reduced the statistical power to identify significant differences between the two training approaches. However, both training approaches reduced the slope index, and the post-training mean and standard deviation values were nearly identical between the two approaches (0.11±0.07 *vs* 0.10±0.07 mmol·L^-1^·min^-1^), indicating that the lack of differences between training approaches regarding the slope index is consistent. Furthermore, the question of whether MCT content and lactate transport capacity are regulated in a dependent and parallel manner in response to chronic exercise has not been fully clarified. While some studies reported that the rate of lactate flux was correlated with the content of MCT1 and MCT4 in the sarcolemmal membrane, other studies showed a dissociation between changes in MCT content and lactate transport activity (for a review, see reference 5). Therefore, the lack of differences between the two approaches in the lactate slope index, even with the twice-a-day approach leading to an acute increase in *MCT1* gene expression, might indicate that *MCT1* gene expression alone does not affect plasma lactate levels. Thus, further studies with longer training periods, more homogeneous samples, and measuring training-induced changes in MCT1 protein content are warranted. However, our findings provide important novel insights regarding the influence of different train-low approaches on *MCT1* gene expression and changes in plasma lactate concentration (39). Our findings indicate that although the acute twice-a-day training results in higher *MCT1* gene expression than the once-daily training, this does not result in changes in plasma lactate kinetics during a graded exercise test.

Our study had some limitations. As previously mentioned, we did not evaluate the MCT1 and MCT4 protein content pre- and post-training in Study 2. Thus, we cannot ascertain whether changes in the plasma lactate curve were related to changes in MCT1 protein content. In addition, plasma lactate concentration does not allow us to infer lactate flux across muscle fibers. Studies measuring muscle lactate concentration and/or lactate production and clearance via lactate tracers will be useful for improving our understanding of training adaptations induced by twice-a-day and once-daily training. The small sample size is another limitation that may have reduced statistical power. The η_p_^2^ can be considered large for the condition factor and condition *vs* moment interaction for *MTC4* gene expression (Study 1), moderate for the moment factor for *MTC4* gene expression (study 1), and moderate for the group factor and group *vs* moment interaction for exercise duration and peak plasma [La] (Study 2). Thus, it is possible that with a larger sample size, we might have detected a significant condition *vs* moment interaction for *MTC4* gene expression and group *vs* moment interaction for exercise duration and peak plasma [La]. A study with a larger sample size may offer further insights into the effects of twice-a-day and once-daily training on these parameters. However, the inherent difficulty in conducting studies involving muscle biopsies and complex training strategies that include long training sessions and dietary manipulation should be considered.

In conclusion, a single session of twice-a-day but not once-daily exercise increases *MCT1* gene expression. Despite the great differences in *MCT1* gene expression, three weeks of both types of training induced similar changes in the lactate slope index, indicating they induce similar changes in the rate of plasma lactate accumulation.
